# Effect of ICU specialist care quality control team management program in continuous renal replacement therapy: A retrospective comparative study

**DOI:** 10.1097/MD.0000000000042710

**Published:** 2025-10-24

**Authors:** Xiaojuan Xu, Min Wang, Yan Wang, Zhijuan Pan, Qiao Liu, Xinyi Shi

**Affiliations:** a ICU, Xianning Central Hospital, Xianning, Hubei, China; b Internal Medicine-Neurology, Xianning First People’s Hospital, Xianning, Hubei, China; c College of Nursing, Guangdong Pharmaceutical University, Guangzhou, Guangdong, China; d Operating Room, Xianning Central Hospital, Xianning, Hubei, China; e Respiratory Medicine Department, PLA Strategic Support Force Characteristic Medical Center, Shenyang, Liaoning, China; f Radiology Department, Interventional Ward, Xianning Central Hospital, Xianning, Hubei, China.

**Keywords:** continuous renal replacement treatment, ICU specialist nursing quality control team, retrospective study, safety management

## Abstract

This study aims to evaluate the effectiveness of the intensive care unit (ICU) specialist care quality control team management of patients treated with continuous renal replacement therapy (CRRT). A retrospective comparative study design was used to compare 2 groups of critically ill CRRT patients in the ICU. Subjects were 519 critically ill CRRT patients admitted to the ICU from January 2018 to December 2021. The control group (n = 265) received routine bedside care management of CRRT, and the experimental group (n = 254) received management by the CRRT specialist quality control group. Outcomes compared included unplanned down rate, unplanned down time, continuous treatment > 24 hours up to standard rate, 72 hours up to standard scheduled down rate, average daily hemodialysis cost, average use time of a single filter, unplanned extubation rate, incidence of catheter-associated bloodstream infection, nursing satisfaction, blood biochemical indexes, and coagulation indexes. The experimental group had a lower unplanned down rate(28.8% vs 42.6%), increased average unplanned down time (20.91 ± 11.21 vs 15.71 ± 7.33), higher continuous treatment > 24 hours up to standard rate (69.0% vs 55.5%), increased 72 hours up to standard scheduled down rate (16.7% vs 8.6%), decreased average daily hemodialysis cost (2947.3 ± 231.22 vs 3508.3 ± 423.22), prolonged average use time of a single filter (39.8 ± 9.47 vs 25.84 ± 7.62), lower unplanned extubation rate (1.9% vs 12.8%), and lower incidence of catheter-related bloodstream infection (0.7% vs 6.8%) compared to the control group (*P* < .05). The experimental group showed higher nursing satisfaction than the control group (92.9% vs 75.1%, *P* < .05). Blood potassium, sodium, blood urea nitrogen, and serum creatinine levels were significantly lower in the experimental group, with improved coagulation index values compared to controls (*P* < .05). ICU nursing quality control team management plan improve the quality of medical care, ensure patients to achieve good therapeutic effect of the goal, worthy of clinical application.

## 1. Introduction

Continuous renal replacement therapy (CRRT) is defined as continuous blood purification therapy for 24 h/d or nearly 24 h/d. This treatment is a general term for methods of replacing damaged kidneys and removing water and solutes continuously and slowly.^[1]^ With the development of medical technology in recent years, CRRT has been used to support the treatment of organ function or in the field of acute and critical illness.^[2]^ In the course of bedside CRRT treatment for intensive care unit (ICU) patients, complications such as extracorporeal circulation and coagulation are prone to occur due to complex disease conditions and coagulation disorders, etc, and unplanned discontinuation is a common problem encountered in the treatment of CRRT in ICU.^[3,4]^ As the most direct implementor in the process of CRRT, the nursing quality of nurses has a very important impact on their unplanned downplay rate.^[5]^ High frequency of downplay will increase the cost of treatment and reduce the therapeutic effect of patients, both of which will eventually lead to more treatment pressure on patients’ families.^[6]^

Based on the cases of bedside CRRT treatment for critically ill patients in our hospital and the personal experience and clinical experience of accompanying medical staff, it is found that there are still many problems to be solved in bedside CRRT treatment for critically ill patients in our department after following the logic of evidence-based nursing and sorting out clues: bedside CRRT does not have a set of unified operation procedures, and it lacks perfect management norms for medical staff; the anticoagulant program of bedside CRRT, the monitoring and evaluation frequency of hemagglutination, and the adjustment of anticoagulant agents are all lacking more unified and reasonable standardized operations^[7]^; ICU nurses have problems in the training of CRRT operation courses, for example, no standardized training courses have been set, let alone the compliance assessment of their operations. In order to effectively solve the quality defect problem of CRRT and greatly reduce the probability of adverse medical events, there must be a set of feasible and specific operation programs. There have been few reports on the effectiveness of the ICU specialist quality control team in the management of bedside CRRT. Therefore, against this background, the objective of this paper is to evaluate the effectiveness of the ICU specialist quality control team in the management of patients treated with CRRT.

## 2. Materials and methods

### 2.1. The selection of research object data

In this study, 519 critically ill patients with CRRT admitted to ICU of our hospital from January 2018 to December 2021 were included. According to different nursing methods, patients with routine bedside CRRT nursing management were set as the control group (265 cases), and the management of CRRT specialist quality control group was set as the experimental group (254 cases). Inclusion criteria: age > 18 years old; bedside CRRT is needed; ICU stay time > 48 hours; sign the relevant consent. Exclusion criteria: incomplete clinical data; in pregnancy or lactation period; and complicated with malignant tumor; inability to tolerate CRRT therapy. This study was approved by the Ethics Committee of Xianning Central Hospital (No. XNCH2018SI) in accordance with the Declaration of Helsinki. All patients or their legal representatives provided written informed consent prior to enrollment.

### 2.2. Therapeutic method

The control group adopted the conventional nursing method, and the supervisor carried out the corresponding nursing before, during and after CRRT loading under the guidance of the doctor combined with their own experience, such as close observation of the changes of vital signs, management of instrument operation, vascular access management, fluid management, complications management, etc. The corresponding experimental group was combined with the treatment of CRRT through the ICU specialized nursing quality control team management program. The specific methods were as follows.

#### 2.2.1. Set up ICU bedside CRRT specialist nursing quality control team

In the experimental group, the ICU nursing quality control team was composed of the leader, technical support staff and team members. The detailed division of tasks was as follows: The team leader was the head nurse of ICU, whose job was to coordinate and communicate with all aspects and manage the team; The deputy head nurse of ICU serves as the teaching group leader, responsible for the teaching and operation of the members of the nursing quality control group of CRRT treatment specialty, and the teaching group leader is also required to assess the members in a certain time range. In addition, the instrument manager is responsible for the quality control and maintenance of the daily work of the CRRT machine, and regularly contact the merchants to calibrate and maintain the CRRT. Finally, the director of the department providing technical support and guidance plus 2 attending physicians. The other part consists of 12 team members, including 1 deputy chief nurse, 7 senior chief nurse, and 1 ICU specialist nurse. Procedures, standards, guidelines, expert consensus and literature related to bedside CRRT treatment were reviewed, and specialized care for bedside CRRT treatment for ICU patients was developed based on the characteristics of critically ill patients.

#### 2.2.2. Formulation of CRRT nursing process and safety management objectives of specialist quality control management team

According to the actual cases in our department and the nursing experience of medical staff, the ICU temporary hemodialysis tube placement process, bedside CRRT treatment order sheet for critical patients, bedside CRRT treatment and nursing record sheet for critical patients, bedside CRRT loading and unloading operation flow and other related workflow were developed. “CRRT Safety Management Objective” is the management standard formulated by the ICU quality control management team with the purpose of “patient safety first.” The specific contents are as follows: Doctor’s order sheet, nursing record sheet with accuracy and completeness, specific implementation method of CRRT standardized process, monitoring means of CRRT catheter infection, CRRT as planned, no CRRT-related adverse events occurred.

#### 2.2.3. The specialist quality control management team standardized the anticoagulation program of bedside CRRT treatment

A crucial point in CRRT nursing is to avoid the occurrence of cardiopulmonary bypass clotting. In order to reduce or even avoid such a situation, the ICU quality control team reviewed the CRRT anticoagulant literature, further improved and standardized the original CRRT anticoagulant program, and actively implemented it, organizing general medical staff to learn and implement the relevant practical content. The biggest difference between this program and the past is that the process of developing anticoagulation program for patients is no longer decided by doctors according to their experience but should be developed by doctors and quality control team together, and in the process of development, patients should be combined with their own situation, so as to achieve 1 case.

#### 2.2.4. Technical support, quality control and emergency handling of CRRT by specialist quality control management team

The details are as follows: first, supervise the catheterization process of doctors to observe whether the catheterization process meets the requirements of the established specifications, such as whether the catheterization process is super guided and whether the catheterization process is the maximum sterile barrier. Second, observe whether the medical staff strictly follow the standardized process during the installation and prefilling. The specific observation points include whether the patients have systemic heparinization and whether the operation process of nurses on and off the machine meets the standard requirements. Third, in addition to the supervision of the work process, team members also need to deal with emergencies. If there is any problem that cannot be solved, the event should be sent to the WeChat group in the form of video or pictures within a certain time, so as to facilitate further discussion and analysis with other members, so as to promote the effective and safe solution of the event.

### 2.3. Statistical analysis

In this study, IBM SPSS Statistics for Windows, Version [25.0] (IBM Corp., Armonk) was used for statistical analysis of the data. For continuous variables, if they conform to normal distribution, parametric test is adopted and expressed as mean ± standard deviation; if they do not conform to normal distribution, nonparametric test is adopted and expressed as median (quartile, IQR). For categorical variables, the percentage of patients in each category was calculated, expressed as n (%). Chi-square test was used for comparing categorical variables between groups, Student *t* test for normally distributed continuous variables, and Mann–Whitney *U* test for non-normally distributed continuous data. Fisher’s exact test was applied when expected cell counts were <5. *P* < .05 was considered statistically significant.

## 3. Result

### 3.1. Comparison of general data between the 2 groups

By comparing the general clinical data of the included control group and the experimental group, it was found that there was no statistical difference in the basic clinical characteristics of the 2 groups of patients (*P* > .05), as shown in Table 1.

**Table 1:** Comparison of general data between the 2 groups.

Characteristics	Control group	Experimental group	*t*/χ2 value	*P*
All patients	(n = 265)	(n = 254)		
Age (yr, *x̄* ± *s*)	60 ± 15	52 ± 18	1.247	.625
Gender (n, %)			0.008	.93
Male	120 (45.3)	116 (45.7)		
Female	145 (54.7)	138 (54.3)		
Clinical diagnosis (n, %)			5.102	.277
Renal failure	103 (38.9)	94 (37.0)		
Multiple injury	68 (25.6)	54 (21.3)		
Poisoning	5 (1.9)	6 (2.4)		
Septic shock	43 (16.2)	60 (23.6)		
CPR (cardiopulmonary resuscitation)	46 (17.4)	40 (15.7)		

CPR = cardiopulmonary resuscitation.

### 3.2. The effect evaluation indexes of CRRT treatment were compared between the 2 groups

By comparing the treatment effect of CRRT between the experimental group and the control group, it was found that compared with the control group, the unplanned down rate, unplanned extubation rate, the incidence of catheter-related bloodstream infection and the average daily hemodialysis cost of patients in the experimental group were all decreased (*P* < .05). However, the compliance rate of 72-hour scheduled deplaning, continuous treatment > 24 hours compliance rate, the average time of unplanned deplaning and the average time of single filter were increased (*P* < .05). The specific results are shown in Table 2.

**Table 2:** The effect evaluation indexes of CRRT treatment were compared between the 2 groups.

Index	Control group	Experimental group	*t*/χ2 value	*P*
All patients	(n = 265)	(n = 254)		
Unplanned lower probability (n, %)	153 (42.6)	109 (28.8)	11.399	.001
72-h planned disembarkation rate (n, %)	31 (8.6)	63 (16.7)	15.018	<.001
Compliance rate of continuous treatment > 24 h (n, %)	147 (55.5)	189 (69.0)	20.376	<.001
Incidence of unplanned extubation (n, %)	31 (12.8)	5 (1.9)	19.091	<.001
Incidence of catheter-associated bloodstream infections (n, %)	18 (6.8)	2 (0.7)	12.623	<.001
Average unplanned downtime (h)	15.71 ± 7.33	20.91 ± 11.21	7.021	.004
Average service time of individual filter (h)	25.84 ± 7.62	39.8 ± 9.47	3.647	.007
Average daily hemodialysis cost of patients (yuan)	3508.3 ± 423.22	2947.3 ± 231.22	6.521	.005

CRRT = continuous renal replacement therapy.

### 3.3. Comparison of nursing satisfaction between 2 groups of patients

The nursing satisfaction of the experimental group was 92.9% (236/254) significantly higher than that of the control group (75.10% (199/265)), the difference between the 2 was statistically significant (*P* = .05; Table 3).

**Table 3:** Comparison of nursing satisfaction between 2 groups of patients.

Groups	n	Quite satisfied	More satisfied	Dissatisfied	Degree of satisfaction
Experimental group	254	182 (71.7)	54 (21.3)	18 (7.1)	236 (92.9)
Control group	265	154 (58.1)	45 (17.0)	66 (24.9)	199 (75.1)
χ2 value					24.501
*P*					<.05

### 3.4. Comparison of therapeutic index results between the 2 groups

The biochemical indexes of blood potassium, blood sodium, blood urea nitrogen and serum creatinine in the observation group were lower than those in the control group, and the differences between the 2 were statistically significant (*P* = .05; Table 4).

**Table 4:** Comparison of therapeutic index results between the 2 groups.

Groups	n	Serum potassium (mmol/L)	Serum sodium (mmol/L)	SCr (μmol/L)	BUN (mmol/L)
Control group	265	4.67 ± 0.12	142.61 ± 4.56	158.73 ± 18.62	10.43 ± 1.58
Experimental group	254	3.98 ± 0.08	134.78 ± 4.13	112.54 ± 11.62	7.96 ± 1.36
*t*		2.798	13.542	4.982	4.547
*P*		<.05	<.05	<.05	<.05

BUN = blood urea nitrogen, SCr = serum creatinine.

### 3.5. Comparison of improvement of coagulation index after nursing between 2 groups

In the comparative investigation of coagulation indexes, the values of the observation group were significantly better than the control group, the difference was statistically significant (*P* < .05; Table 5).

**Table 5:** Comparison of improvement of coagulation index after nursing between 2 groups.

Groups	n	PT	APTT	TT
Control group	265	10.35 ± 1.67	26.48 ± 3.18	14.92 ± 1.43
Experimental group	254	15.74 ± 1.23	36.23 ± 2.78	21.63 ± 1.87
*t*		3.948	8.379	6.216
*P*		.009	.003	.008

APTT = activated partial thromboplastin time, PT = prothrombin time, TT = thrombin time.

## 4. Discussion

The use of CRRT therapy plays an important role in the therapeutic effect and prognosis of ICU patients. However, current studies have shown^[7]^ that there are still many factors that can lead to unplanned discontinuation during the implementation of CRRT, which not only greatly affects the therapeutic effect of patients. It also increases the psychological and economic burden on patients and their families, so the ICU quality control nursing team should pay more attention to these problems in the process of work and strive to avoid unnecessary harm to patients and their families. The purpose of this study was to investigate the effect of ICU care quality control team on patients with CRRT. After data collection and analysis, we found that the ICU specialized nursing quality control team management plan is conducive to patient rehabilitation and process specification in CRRT execution.

When interpreting our findings, it is important to consider the causal relationship between the ICU specialist nursing quality control team intervention and the observed outcomes within the framework of target trial emulation (TTE). As highlighted in recent methodological advances in retrospective comparative research, TTE provides a structured approach to mimic the design elements of a randomized controlled trial using observational data. This framework helps address potential confounding factors and selection biases that are inherent in retrospective comparative studies such as ours.

In our study context, numerous confounding variables could influence the relationship between the quality control team intervention and CRRT outcomes. These include patient severity at baseline, comorbidities, variations in physician practice patterns, staffing ratios, and concurrent changes in ICU protocols during the study period. While we have demonstrated comparable baseline characteristics between our experimental and control groups, unmeasured confounders may still exist. Future research could employ more advanced statistical methods such as propensity score matching, inverse probability weighting, or instrumental variable analysis to better emulate a target trial and strengthen causal inference.

The management plans of ICU specialized nursing quality control team formulated in this study include: standardized operation process of catheterization was developed and implemented,^[8]^ which effectively reduced high frequency machine alarm caused by improper position and blocked blood flow during catheterization; second, the process of standardizing the use of CRRT anticoagulant by medical staff has been formulated to effectively reduce filter alarm and even unplanned agglutination caused by lack of anticoagulant.^[9]^ Thirdly, the quality control team members were given strict teaching and training, which strictly required the regularization of boarding and disembarkation in the process of CRRT treatment, in order to effectively reduce the occurrence of unplanned disembarkation events caused by the medical staff’s unskilled operation of the machine.

Generally speaking, there are 2 preconditions for the occurrence of catheter-related bloodstream infection. One is external, which includes but is not limited to the environment at the time of catheterization, the whole process of catheterization operation, the performer’s own technical level, the position of catheterization and the way of tube sealing. One is internal, and generally only external factors can be controlled. In reality, effectively reducing the occurrence of external risk factors itself can meet the requirements of reducing the incidence of bone dry bloodstream infection in catheterization.^[10–12]^ In this context, this study tested and collected data on the management plan of the CRRT specialist quality control team, and the data analysis results proved that the implementation of this management plan can effectively reduce the incidence of catheter-related bloodstream infections in patients requiring hemodialysis. Meanwhile, the collected data also showed that, The quality control team management plan can implement the technical specifications of the catheterization process of medical staff, such as monitoring whether the implementation of hand hygiene and skin disinfection system, whether to ensure the establishment of maximum sterile barrier during the catheterization operation; The operation specification of the quality control team suggests that puncture guided by B-ultrasound can not only effectively shorten the puncture time, but also improve the success rate of each puncture. Once the medical staff can strictly implement this program, the possibility of bacterial invasion of the patient’s body can be truly reduced, and the incidence of infection can be effectively reduced. This data analysis result was consistent with previous research results.^[13–15]^ In addition, in the improvement management plan for the specialized quality control team, there are strict requirements for the onboard and off-board operation of medical staff, including the disinfection method of catheter interface, which can effectively reduce a series of infection events caused by the failure of catheter interface disinfection, and effectively reduce the incidence of infection of patients.

Most patients undergoing CRRT treatment need to undergo hemodialysis. Therefore, in the management plan of the specialized quality control team, strict and standardized teaching training should be provided to medical staff, especially nurses, to ensure that they can correctly use hemodialysis catheters in the actual operation process and complete the follow-up maintenance of catheters. This method reduces the chance of thrombosis in the hemodialysis tube, keeps the catheter unblocked, and reduces the chance of catheter infection.^[16]^ The operation guidance of the quality control team for medical staff also includes the behavior of puncture under B-ultrasound positioning, which can reduce iatrogenic extubation caused by wrong placement of tubes and wrong selection of blood vessels.^[17,18]^ The above discussions are all about the quality control and management during the implementation of CRRT treatment in the management plan of the quality control team of the specialty. All these plans can truly avoid the phenomenon of unplanned extubation caused by improper medical care. Therefore, the plan requires the members of the quality control team to be on duty for 24 hours, which can not only supervise the smooth implementation of the plan. To reduce the probability of unplanned disengagement caused by improper operation of medical staff, it is an inevitable choice to timely deal with various emergencies in the process of CRRT.

In addition, in this study, the experimental group of patients in the serum creatinine, urea nitrogen, blood potassium, sodium and other indicators of good control, can further indicate that the quality of ICU care quality control team management program can not only guarantee the continuous effect of kidney replacement therapy, but also effectively remove harmful substances, ensure the anticoagulant effect, for improving the quality of care and family satisfaction, a stable and sound nurse–patient relationship is of great significance.^[19,20]^

It is worth noting that we observed multiple positive outcomes across various domains including biochemical parameters, anticoagulation indices, and patient satisfaction. From a TTE perspective, this constellation of positive findings strengthens the inference that our intervention had a genuine causal effect rather than simply representing statistical artifacts or chance findings. However, the retrospective nature of our study design limits our ability to definitively establish causality. A prospective stepped-wedge cluster-randomized trial would be the ideal next step to validate our findings within a more rigorous experimental framework.

Additionally, our findings should be interpreted in light of potential time-varying confounding that occurs when the relationship between exposure and outcome is affected by factors that change over time and are themselves affected by prior exposure. Advanced statistical methods such as marginal structural models or g-estimation could be employed in future studies to address such complex confounding scenarios, further strengthening the causal interpretation of the relationship between specialized nursing quality control teams and CRRT outcomes.

To sum up, nursing quality control group management is to deal with the CRRT treatment process of the new management, its primary purpose is to ensure that managers and their subordinates work results and original plan to match, effective management and correction to reduce the implementation of deviation, and finally achieve the realization of management objectives in the process of treatment. While our findings support the effectiveness of this approach, applying the TTE framework helps identify methodological limitations and points to opportunities for strengthening causal inference in future research on specialized nursing interventions in critical care settings.

## 5. Conclusion

ICU nursing quality control team management plan from the perspective of patients, effectively strengthen the quality management of CRRT nursing, comprehensive supervision of medical staff catheterization evaluation, site selection, process implementation, process quality control of these processes, so as to improve the quality of medical care, ensure patients to achieve good therapeutic effect of the goal, worthy of clinical application.

## Acknowledgments

All authors contributed to the study conception and design, data collection, analysis, manuscript preparation, and agreed to be listed as authors. All individuals acknowledged have given permission to be named.

## Author contributions

**Data curation:** Qiao Liu.

**Formal analysis:** Qiao Liu.

**Funding acquisition:** Xinyi Shi.

**Project administration:** Xinyi Shi.

**Resources:** Min Wang, Zhijuan Pan.

**Software:** Yan Wang.

**Supervision:** Xinyi Shi.

**Visualization:** Xinyi Shi.

**Writing – original draft:** Xiaojuan Xu, Xinyi Shi.

**Writing – review & editing:** Xinyi Shi.
